# *Panax notoginseng* saponins prevent dementia and oxidative stress in brains of SAMP8 mice by enhancing mitophagy

**DOI:** 10.1186/s12906-024-04403-7

**Published:** 2024-04-04

**Authors:** Yingying Yang, Wenya Chen, Zhenmei Lin, Yijing Wu, Yuqing Li, Xing Xia

**Affiliations:** 1 School of Pharmacy, Guangxi University of Chinese Medicine, Nanning, 530200 China; 2 Key Laboratory of TCM Neuro-metabolism and Immunopharmacology of Guangxi Education Department, Guangxi University of Chinese Medicine, Nanning, 530200 China; 3 School of Public Health and Management, Guangxi University of Chinese Medicine, Nanning, 530200 China

**Keywords:** *Panax notoginseng* saponins, Dementia, Mitochondrial autophagy, PINK1-Parkin

## Abstract

**Background:**

Mitochondrial dysfunction is one of the distinctive features of neurons in patients with Alzheimer’s disease (AD). Intraneuronal autophagosomes selectively phagocytose and degrade the damaged mitochondria, mitigating neuronal damage in AD. *Panax notoginseng* saponins (PNS) can effectively reduce oxidative stress and mitochondrial damage in the brain of animals with AD, but their exact mechanism of action is unknown.

**Methods:**

Senescence-accelerated mouse prone 8 (SAMP8) mice with age-related AD were treated with PNS for 8 weeks. The effects of PNS on learning and memory abilities, cerebral oxidative stress status, and hippocampus ultrastructure of mice were observed. Moreover, changes of the PTEN-induced putative kinase 1 (PINK1)-Parkin, which regulates ubiquitin-dependent mitophagy, and the recruit of downstream autophagy receptors were investigated.

**Results:**

PNS attenuated cognitive dysfunction in SAMP8 mice in the Morris water maze test. PNS also enhanced glutathione peroxidase and superoxide dismutase activities, and increased glutathione levels by 25.92% and 45.55% while inhibiting 8-hydroxydeoxyguanosine by 27.74% and the malondialdehyde production by 34.02% in the brains of SAMP8 mice. Our observation revealed the promotion of mitophagy, which was accompanied by an increase in microtubule-associated protein 1 light chain 3 (LC3) mRNA and 70.00% increase of LC3-II/I protein ratio in the brain tissues of PNS-treated mice. PNS treatment increased Parkin mRNA and protein expression by 62.80% and 43.80%, while increasing the mRNA transcription and protein expression of mitophagic receptors such as optineurin, and nuclear dot protein 52.

**Conclusion:**

PNS enhanced the PINK1/Parkin pathway and facilitated mitophagy in the hippocampus, thereby preventing cerebral oxidative stress in SAMP8 mice. This may be a mechanism contributing to the cognition-improvement effect of PNS.

**Supplementary Information:**

The online version contains supplementary material available at 10.1186/s12906-024-04403-7.

## Introduction

Alzheimer’s disease (AD) is an age-related neurodegenerative disease characterized by cognitive and memory dysfunction and dementia; it has a significant impact on patients’ quality of life [1]. The risk of AD increases considerably with advanced age. More than 50 million people worldwide currently live with AD, and this number is expected to exceed 152 million by 2050 [2]. Only 5 drugs, namely donepezil, rivastigmine, galantamine, tacrine, and memantine, are currently approved by the US Food and Drug Administration to treat AD [3]. These drugs only show some efficacy in alleviating cognitive deficits in patients with mild-to-moderate AD and cannot reverse its progression [4, 5]. It is evident that there exists a necessity to enhance the advancement of pharmaceuticals aimed at the treatment of AD.

The main pathological features of AD include the formation of senile plaques with a core of β-amyloid (Aβ) deposits outside neurons, intracellular tangles of neurogenic fibers with a core of hyperphosphorylated Tau, and neuronal loss [6]. A large body of evidence also suggests that mitochondrial dysfunction, oxidative stress, and autophagy are involved in the pathogenesis of AD [7–9]. For example, Aβ deposition and accumulation in the brain can lead to oxidative stress, promoting and accelerating AD onset. Healthy mitochondria can provide sufficient energy for neuronal activity and protect neurons by minimizing mitochondria-related oxidative damage [10]. Mitochondrial autophagy, also known as mitophagy, plays a key role in maintaining neuronal health, whereas autophagosomes remove damaged mitochondria to maintain homeostasis and normal cellular activities in the brain [11–13]. Mitophagy is impaired in the hippocampi of patients with AD and in the brains of mice with AD, leading to ineffective clearing of the damaged mitochondria. Further, the accumulation of damaged mitochondria causes Tau protein to aggregate; elevated levels of Aβ and phosphorylated Tau increase reactive oxygen species production and abnormal mitochondrial division, ultimately exacerbating mitochondrial damage [14]. PTEN-induced putative kinase 1 (PINK1)/Parkin is an important signaling pathway associated with mitosis [15] and is key in controlling mitophagy [16]. Activating the PINK1/Parkin pathway enhances mitophagy, inhibits apoptosis, and protects neuronal cells [17], thus leading to mitophagy; therefore, it could be a potential therapeutic strategy in treating AD.

*Panax notoginseng* is a widely used traditional Chinese medicine for treating cardiovascular and cerebrovascular diseases [18–20]. Clinically, it reduces cognitive dysfunction and brain damage in ischemic encephalopathy and acute stroke [21–23]. Its main active ingredient is *P. notoginseng* saponins (PNS), which have been formulated as Chinese patent medicine such as *P. notoginseng* Tong Shu capsules and Xueshuantong capsules and widely used clinically to treat dementia [24]. PNS play a protective role in learning dysfunction in the senescence-accelerated mouse prone 8 (SAMP8) mice [25] and APP/PS1 transgenic mice [26], and can inhibit AD-like symptoms of worm paralysis in *Caenorhabditis elegans* [27]. Many mechanisms including precluding Aβ generation and deposition [25], skinhead-1 pathway activation [27], antioxidative mechanism [28], circRNAs regulation mechanism [29], are involved in the therapeutic effect of PNS. SAMP8 model shows an early decline in learning and memory [30], as well as several brain abnormalities comparable with those seen in AD, such as increased Aβ deposition, tau phosphorylation, and oxidative stress [31]. SAMP8 mice present a demented phenotype, which relates to the level of autophagy in the nervous system [32]. PNS exerts neuroprotective effects in SAMP8 mice, thereby alleviating cognitive dysfunction [33]. It also exerts therapeutic effects by promoting autophagy [34, 35], and its mechanism of enhancing mitophagy is a key pathway attributed to its mechanism of action [36]. PNS can promote mitophagy and alleviate cerebral ischemia–reperfusion injury in rats [37]. Notoginsenoside R1, one of the key components of PNS, can alleviate diabetic complications by enhancing PINK1-dependent mitochondrial autophagy [38]. Our previous study found that PNS could protect PC12 cells against Aβ-induced injury by promoting parkin-mediated mitophagy in vitro [39]. PNS exerts excellent neuroprotective effects and decreases neurological deficits, thereby improving cognitive dysfunction. However, the specific mechanisms of action and targets of PNS in enhancing cognitive function in vivo need to be further explored. Based on our previous observations, we proposed the following hypothesis: PNS can alleviate oxidative stress and regulate mitochondrial autophagy in the brain, thus improving the learning memory abilities of AD. To explore this hypothesis, the role of PNS in preventing oxidative stress and improving mitophagy in the hippocampi of SAMP8 mice with dementia were investigated. The findings provide an important basis for its use in treating AD.

## Materials and methods

### Preparation of ***P. notoginseng*** saponins

PNS was purchased from Manster Bio-Technology Co., Ltd, (Chengdu, China, Lot No. MUST-18,070,601), which prepared PNS in compliance with the Chinese Pharmacopoeia. The quality of PNS meets stipulation of Chinese Pharmacopoeia, with 14.5% notoginsenoside R1, 28.0% ginsenoside Rg1, and 27.7% ginsenoside Rb1 as the main components (Chinese Pharmacopoeia 2020 Edition). Both PNS and Huperzine A (Cisen Pharmaceutical Co., Ltd, Shandong, China) were administered intragastrically after homogeneous dispersion with distilled water.

### Animals and treatment

The senescence-accelerated mouse (SAM) is a rapid aging mouse strain selectively bred from the AKR/J line of mice. It is divided into the P strain of senescence-accelerated mice prone (SAMP), which is characterized by a short lifespan and pronounced Aβ pathology; the R strain (SAMR), which is anti-aging and has a normal mouse aging process, and is often used as a normal control for SAMP [40]. Three-month-old male SAMR1 (*n* = 10) and SAMP8 (*n* = 40) mice were purchased from the Department of Medicine, Peking University (Beijing, China), housed under standard conditions (24 ± 2 °C, 12-h light/dark cycle, 60 ± 10% humidity), and provided free access to food and water. All animal-handling procedures were approved by the Animal Ethics Committee of Guangxi University of Traditional Chinese Medicine and conformed to the relevant regulations of the National Experimental Animal Welfare Ethics. All experiments were performed following ARRIVE guidelines 2.0 for reporting animal research.

After 4 weeks of feeding (approximately 4 months of age), the rapidly aging SAMP8 mice were treated according to previous study with some adjustments [33]. SAMP8 mice were randomly divided into a Model group (Model), Huperzine A group (Huperzine A, treated with 0.3 mg/kg of Huperzine A), PNS high-dose group (PNS-Hd, treated with 100 mg/kg of PNS), and PNS low-dose group (PNS-Ld, treated with 50 mg/kg of PNS), with each group consisting of 10 animals, Normal-aging SAMR1 mice served as the control group (Control). Animals in the control and model groups were administered distilled water similarly, once daily for 8 weeks. After completing the Morris water maze (MWM) behavioral experiment, mice were starved for 12 h and anesthetized with an intraperitoneal injection of 60 mg/kg phenobarbital sodium (Fujian Mindong Rejuenation Pharmaceutical Co., Ltd, Fujian, China, Lot No. 180,302). The blood was collected under anesthesia in a dissection room, following which, mice were sacrificed by decapitation and dissected to obtain the brain and hippocampal tissues. Samples were labeled with consecutive numbers by a researcher who had no role in conducting the assays. All investigators only knew the sample number and were unaware of group allocations.

### MWM test

After 8 weeks of drug administration, MWM (BW-MMMIOI, Shanghai Biowill Co., Ltd, Shanghai, China) was used for the orientation-navigation test and probe test [41]. The MWM apparatus consisted of a circular pool with a radius of 100 cm and height of 50 cm, 2/3 of which was filled with water and black food coloring dye (22 ± 1 °C), and a circular platform with a radius of 5 cm and height of 29 cm. Two investigators, who were unaware of the group allocation of mice, conducted the MWM test. In the orientation-navigation test (days 1–5), each mouse was trained to find the platform hidden 1 cm under the water within 60 s. The swimming activity of mice was recorded with a camera placed on top of the water maze and the escape latency time for mice to successfully land on the platform was measured using the ANY-maze system (Stoelting Co., IL, USA). The mice that did not successfully find the platform were guided to it and held there for 10 s, with an escape latency of 60 s. During the probe test (day 6), the platform was removed and the animals were placed in quadrant 2, which was opposite to the target quadrant (quadrant 4, the quadrant where the platform was located). The swimming time and trajectory of mice in the quadrant of the original platform within 120 s were recorded and the percentage of swimming time and path length in the target quadrant to the total time and path length, respectively, was calculated.

### Determination of glutathione peroxidase (GSH-Px), superoxide dismutase (SOD), glutathione (GSH), and malondialdehyde (MDA) levels

Frozen brain tissues were taken and precooled phosphate-buffered saline (PBS) was added to obtain a 10% brain-tissue homogenate after homogenization at 4 ºC and centrifugation at 3000 rpm for 15 min. The supernatant was assayed for GSH-Px and SOD activities and GSH and MDA levels using the corresponding assay kits (Nanjing Jiancheng Biotechnology Co., Ltd, Nanjing, China). One unit of GSH-Px activity was defined as the depletion of 1 μmol/L of GSH in per mg protein per min, deducting the effect of nonenzyme-catalyzed reaction, and results were expressed as U/mg protein. SOD activity was also expressed as U/mg protein, and one unit of SOD was defined as the amount of protein inhibiting nitro-blue tetrazolium reduction by 50%.

### Determination of 8-hydroxydeoxyguanosine (8-OHDG) levels

To determine 8-OHDG levels, the 10% brain-tissue homogenate was repeatedly freeze-thawed twice for adequate lysis and centrifuged at 4 ℃ at 5000 rpm for 5 min. The level of 8-OHDG in the supernatant was determined using an 8-OHDG enzyme-linked immunosorbent assay kit according to the manufacturer’s instructions (Cusabio Co., Ltd, Wuhan, China). The total protein concentration in the supernatant was determined and the level of 8-OHDG in the brain tissue is expressed as the mass of 8-OHDG/mg of tissue protein.

### Transmission electron microscopy

Brain tissues fixed in 2.5% glutaraldehyde solution were rinsed three times with PBS and fixed overnight at 4 °C using 1% osmium tetroxide. The tissues were rinsed 3 times with PBS and subjected to gradient dehydration, infiltration, embedding, polymerization, and slicing. Ultrathin sections were double-stained with 2% uranyl acetate and lead citrate. Morphological changes of cell ultrastructure in the hippocampus were observed and photographed using a transmission electron microscope (H-7650, Hitachi. Ltd., Tokyo, Japan).

### Reverse transcription–polymerase chain reaction (RT-qPCR)

Nine frozen hippocampi were randomly selected from each group, and total RNA was extracted with Trizol, followed by its purity and concentration measurements. Total RNA was reverse-transcribed using the RevertAid First Strand cDNA Synthesis Kit (Thermo Fisher Scientific Inc., MA, USA). Real-time PCR was performed using the SYBR Green kit (Roche Inc., Basel, Switzerland). The PCR amplification conditions were as follows: pre-denaturation at 95 ℃ for 10 min, followed by 40 cycles of denaturation at 95 ℃ for 15 s, annealing at 55 ℃ for 15 s, and an extension of 60 s. The Ct values of the target genes were normalized using β-actin as the internal reference, and their relative expressions were calculated using the 2^−ΔΔCt^ method. Primer sequence information is shown in Table 1.

**Table 1:** Primer Sequence

Gene	Forward Primer (5′-3′)	Reverse Primer (5′-3′)
LC3-I	ATGGTCTACGCCTCCCAAGA	CAGCCAGCACCCAAAAGAG
P62	AGCTGCTTGGCTGAGTGTTAC	CAATTTCCTGAAGAATGTGGG
PINK-1	TCTCAAGTCCGACAACATCCTT	ATTGCCACCACGCTCTACAC
Parkin	CGGTCATTCTGGACAGACAGTAA	TGCAGGTGCCACACTGAACTC
NDP52	CGCTACCAGTTCTGCTATGTGGA	TCATGGAGGTCAGCGTACTTGTC
OPTN	GGACAGCCCTTGTGAGACCC	TCAAATCGCCCTTTCATAGC
β-actin	CATCCGTAAAGACCTCTATGCCAAC	ATGGAGCCACCGATCCACA

### Western blotting

To obtain sufficient protein concentration for western blotting, hippocampi from each group were randomly paired to obtain five pooled samples. Proteins were extracted by adding radioimmunoprecipitation assay buffer (Beyotime Biotech. Inc., Shanghai, China) to lyse fully, and their concentrations were determined using a protein quantification kit (Nanjing Jiancheng Biotechnology Co., Ltd, Nanjing, China). Proteins were separated using sodium dodecyl sulfate–polyacrylamide gel electrophoresis (Beyotime Biotech. Inc., Shanghai, China), transferred to polyvinylidene difluoride membranes (Beijing Solarbio Science & Technology Co., Ltd, Beijing, China), and blocked with 5% skim milk for 2 h. The membranes were then incubated overnight at 4 °C with the primary antibodies: LC3 (1:1000, Cell Signaling Technology. Inc., MA, USA, Lot No. 0003), PINK1 (1:300, Abcam. Inc., Cambridge, UK, Lot No. GR3188319-1), Parkin (1:600, Abcam. Inc., Cambridge, UK, Lot No. GR3172902-1), NDP52 (1:600, Cell Signaling Technology. Inc., USA, Lot No. 0002), and OPTN (1:100, Abcam. Inc., MA, UK, Lot No. GR271296-16). The PVDF membranes were cut into long strips based on protein molecular weights, then membrane strips were washed with Tris-buffered saline containing Tween 20 and incubated with peroxidase-conjugated goat anti-rabbit IgG (Boster Biological Technology Co., Ltd, Wuhan, China, Lot No. BST14G29C54) for 1 h, and then imaged using an enhanced chemiluminescence (ECL) reagent kit (Beyotime Biotech. Inc., Shanghai, China). The bands were quantified using ImageJ, and the relative abundance of the target protein was normalized using β-actin as an internal reference.

### Statistical analysis

Data are presented as the mean ± standard deviation (SD), and statistical analyses were performed using GraphPad Prism 9.0 (GraphPad, La Jolla, CA). One-way analysis of variance (ANOVA) followed by Tukey’s post hoc test. *P* < 0.05 was considered statistically significant.

## Results

### PNS improves learning memory in SAMP8 dementia model mice

**Fig. 1:**
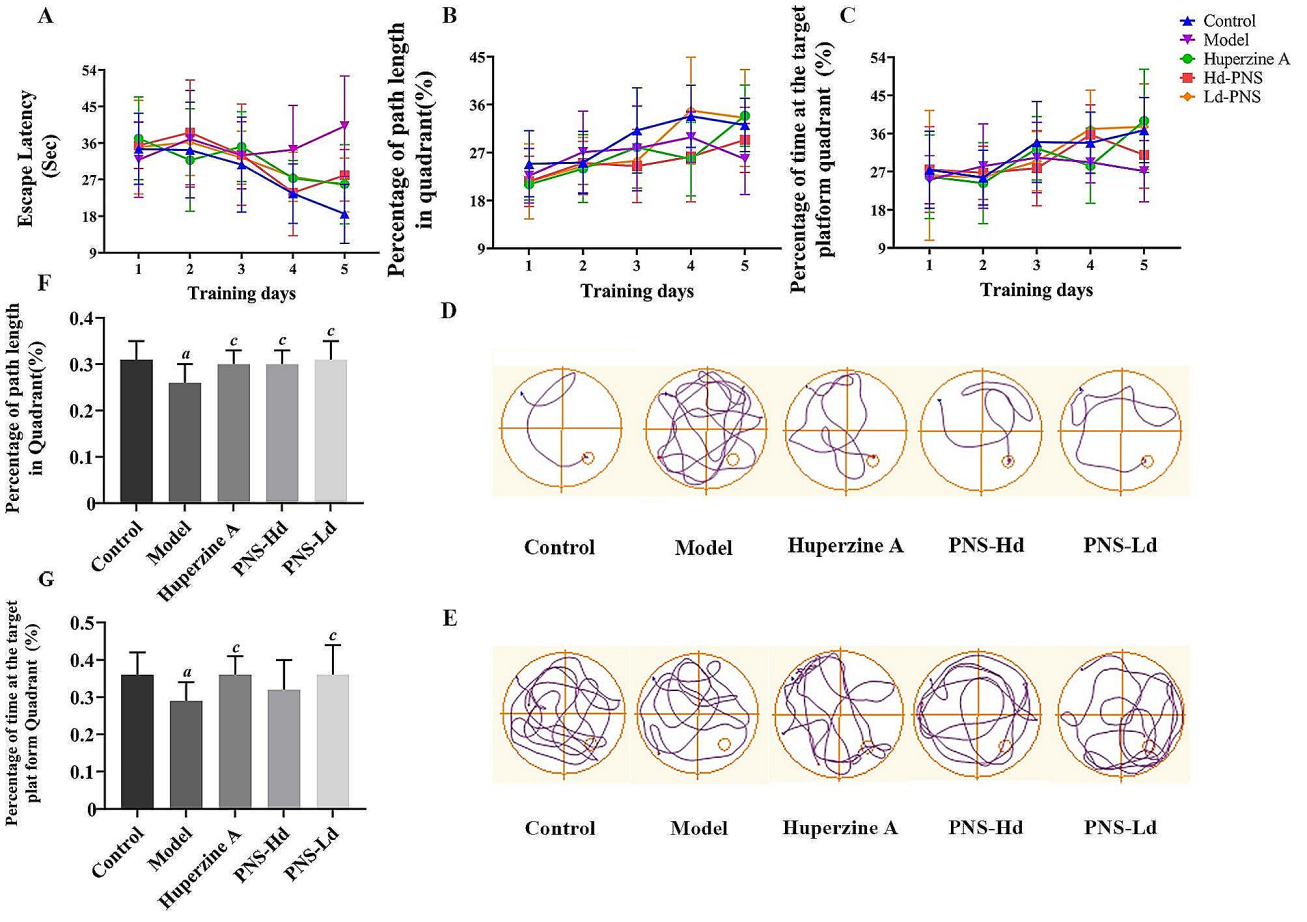
*Panax notoginseng* saponins improve learning and memory ability in SAMP8 mice, *n* = 10. **A** Escape latency in the orientation-navigation test. **B** Platform quadrant distance percent ratio in the orientation-navigation test. **C** Animal platform quadrant dwell time percent ratio in the orientation-navigation test. **D** Swimming trajectory of mice on the 5th day of the orientation-navigation test. **E** Swimming trajectory of mice in the probe test. **F** Probe test platform quadrant distance percentage ratio. **G** Probe test platform quadrant time percentage ratio. ^*a*^*P* < 0.05, ^*b*^*P* < 0.01 compared with the control group, ^*c*^*P* < 0.05, ^*d*^*P* < 0.01 compared with the model group

The MWM test was used to evaluate the learning and memory abilities of SAMP8 mice. No significant differences (*P* > 0.05) were found in the dwell time percentage and path length percentage in the platform quadrant between groups during 1–3 days of the orientation-navigation test. On days 4–5, the escape latency increased significantly (Day 4 model group: 34.35 ± 10.89 s, *P* < 0.05 ; Day 5 model group: 40.14 ± 12.36 s, *P* < 0.01, Fig. 1A), and the path length percentage (Day 5 model group: 25.80 **±** 6.66%, Fig. 1B) and dwell time percentage (Day 5 model group: 27.11 ± 7.23%, *P* < 0.01, Fig. 1C) at the platform quadrant reduced significantly in mice in the model group compared with that of mice in the control group. PNS-treated mice had considerably shorter escape latency time (Day 4 PNS-Hd group: 23.77 **±** 10.57 s, *P* < 0.05 ; Day 5 PNS-Hd and PNS-Ld group: 28.05 ± 6.35 s, *P <* 0.05, 25.65 **±** 6.65 s, *P* < 0.01) and a significantly longer dwell time percentage in the platform quadrant (Day 4 PNS-Hd group and PNS-Ld group: 35.71 **±** 7.05 s, *P* < 0.05, 37.08 **±** 9.14 s, *P* < 0.05; Day 5 PNS-Ld: 37.61 ± 10.10 s, *P* < 0.01) compared with that of mice in the model group. In the probe test performed on the 6th day, mice in the model group showed significantly less swimming time and path length in the target quadrant compared with those in the control group (*P <* 0.05). The activity trajectories of mice did not indicate a preference for the target quadrant and were less likely to cross the original platform area (Fig. 1D-E). Compared with the model group (percentage of path length: 26.0 ± 4.0%; percentage of dwell time: 29.0 ± 5.0%), the percentage of path length in the target quadrant increased considerably in the PNS-Ld group (31.0 ± 4.0%, *P* < 0.05 ) and PNS-Hd group (30 ± 3.0%, *P* < 0.05, Fig. 1F), and the percentage of dwell time in the target quadrant also increased significantly in the PNS-Ld group (36.0 ± 8.0%, *P* < 0.05, Fig. 1G).

### PNS attenuates oxidative stress in the brain of SAMP8 mice

**Fig. 2:**
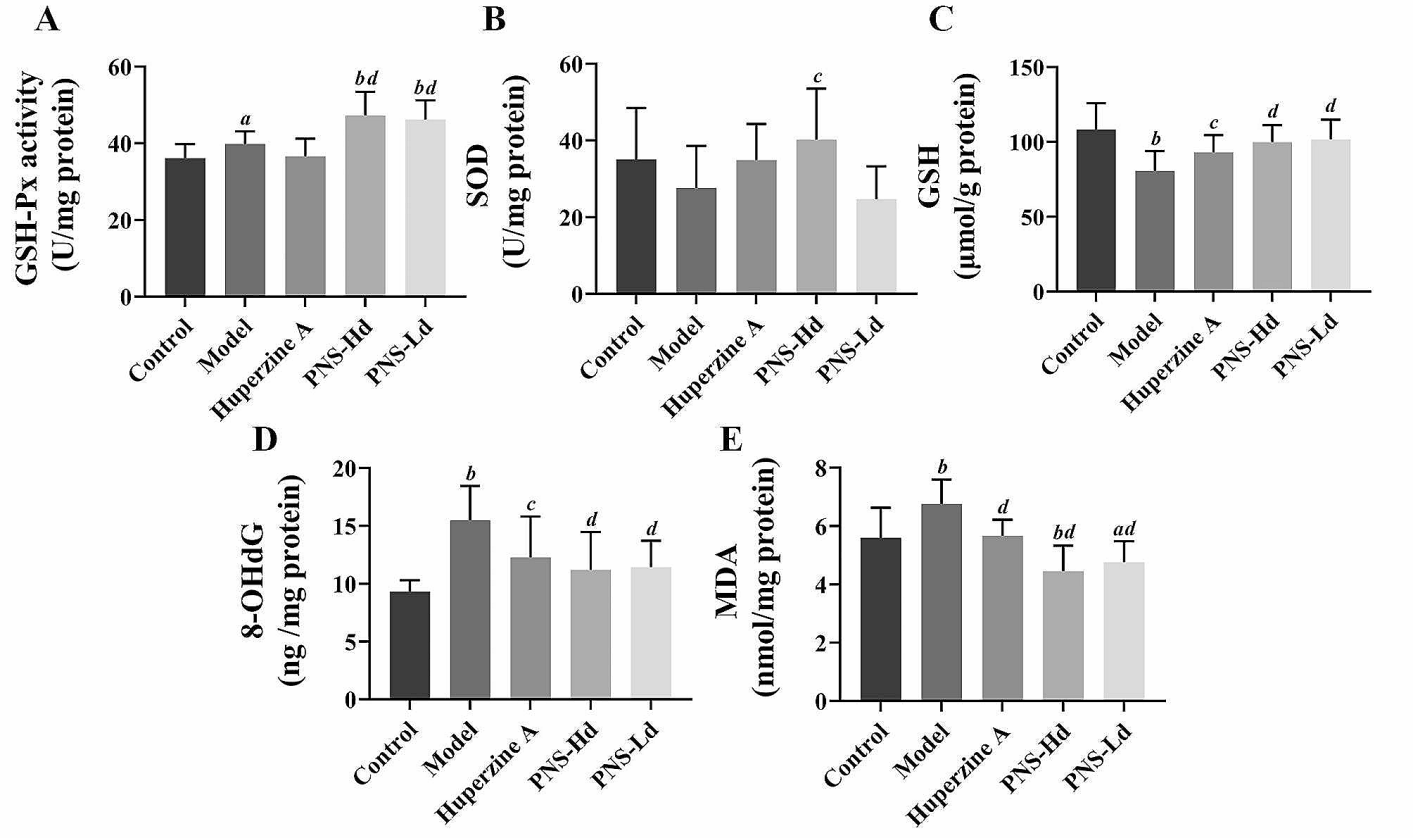
*Panax notoginseng* saponins attenuate oxidative stress damage in the brains of SAMP8 demented mice, *n* = 10. **A** GSH-PX activity, **B** SOD activity, **C** GSH content, **D** 8-OHdG content, and **E** MDA content in mouse brain. ^*a*^*P* < 0.05, ^*b*^*P* < 0.01 compared with the control group, ^*c*^*P* < 0.05, ^*d*^*P* < 0.01 compared with the model group

GSH-Px and SOD activities, and GSH, MDA, and 8-OHDG levels were determined to illustrate the oxidative stress status in the brain of SAMP8 mice. GSH-Px activity in the model group (39.82 **±** 3.31 U/mg prot protein, Fig. 2A) was higher than that in the control group (*P* < 0.05) and was further increased after treatment with high- and low- dose PNS (PNS-Hd: 47.25 ± 6.21 U/mg protein, *P* < 0.01; PNS-Ld: 46.22 **±** 4.98 U/mg protein, *P* < 0.01). The SOD activity decreased slightly in model mice (27.64 ± 10.93 U/mg protein), but increased significantly in mice administered the high doses of PNS (40.23 ± 13.3 U/mg protein, *P* < 0.05; Fig. 2B). GSH levels in model mice (80.72 **±** 13.18 μmol/g protine) were considerably lower than in the controls (*P* < 0.01), and treatment with high- and low-dose PNS significantly increased GSH level 99.94 **±** 11.18 and 101.64 **±** 13.20 μmol/g protine in these groups (*P* < 0.01, Fig. 2C). 8-OHDG and MDA levels significantly increased to 15.50 ± 2.97 ng/mg protein and 6.76 ± 0.84 nmol/mg protein in model mice (*P* < 0.01 and *P* < 0.05) but decreased significantly to 11.20 ± 3.28 ng /mg protein and 4.46 ± 0.87 nmol/mg protein in high-dose PNS treated mice compared with that in model mice (*P* < 0.01; Fig. 2D-E). These results indicated that PNS improves the antioxidant capacity and reduces oxidative stress in the brain of SAMP8 mice.

### PNS enhances autophagy in the hippocampi of SAMP8 mice

**Fig. 3:**
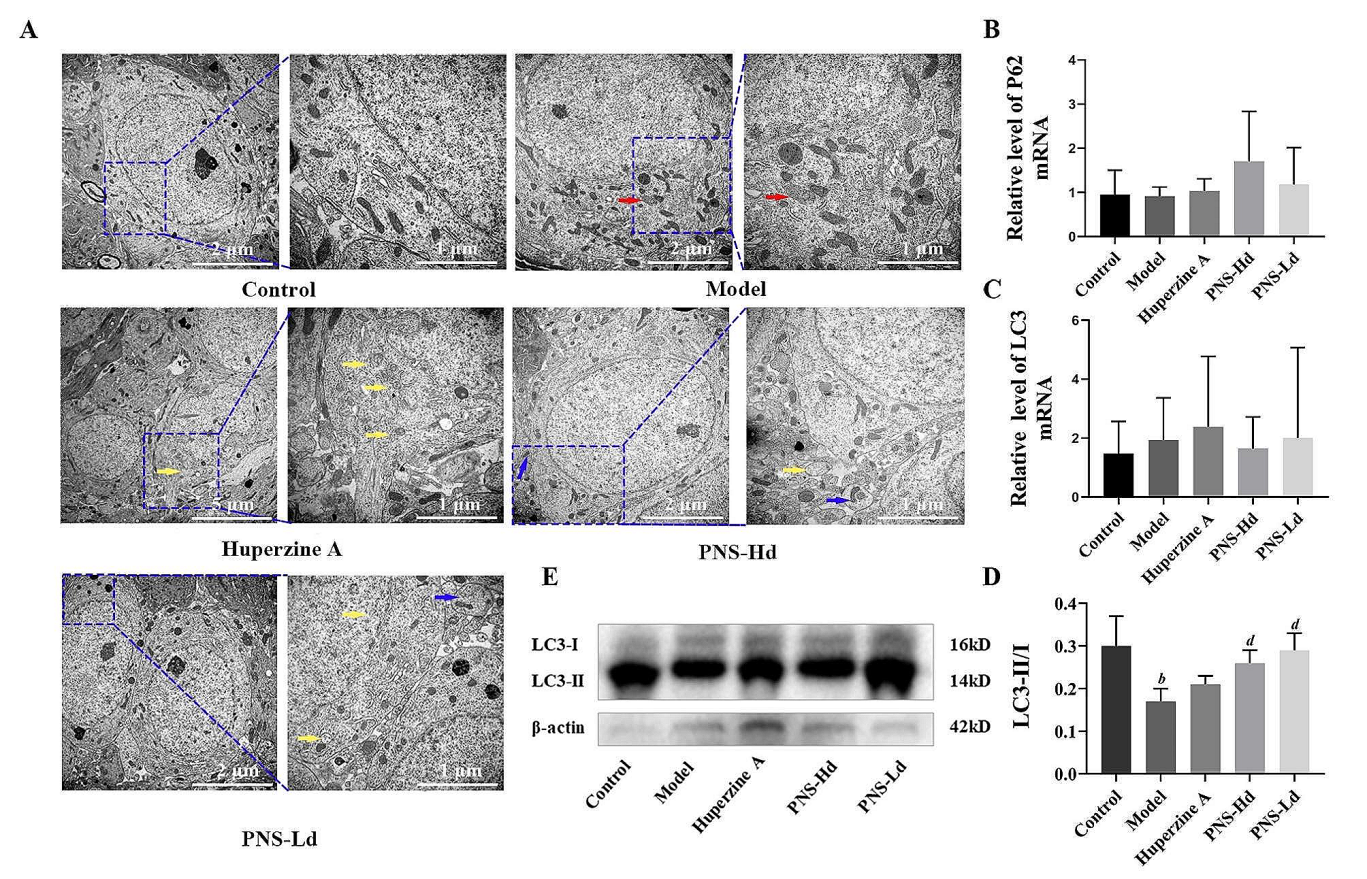
*Panax notoginseng* saponins enhance the level of autophagy in hippocampi of SAMP8 demented mice. **A** Red arrows show transmission electron micrographs of damaged mitochondria, yellow arrows show autophagosomes and blue arrows show autophagosomes containing damaged mitochondria. **B**-**C** LC3 mRNA and P62 mRNA expression in mouse hippocampus, *n* = 9. **D** Western blot of autophagy-related protein LC3 in the mouse hippocampus. **E** LC3-II/I protein ratio in mouse hippocampus, *n* = 5; ^*a*^*P* < 0.05, ^*b*^*P* < 0.01 compared with the control group, ^*c*^*P* < 0.05, ^*d*^*P* < 0.01 compared with the model group

The ultrastructure of hippocampal cells was observed using transmission electron microscopy. Mitochondria were mostly long and oval in the hippocampus obtained from the control group; they had clear cristae and intact structure without overaggregation. In samples from model mice, mitochondria were aggregated around the nucleus of the cells; some were swollen and ruptured and had an obscure structure of the bilayer membrane and scattered endoplasmic reticulum and lysosome vesicles. However, most of the mitochondrial bilayers in the hippocampi of PNS-treated mice were structurally intact with neatly aligned cristae. Mitochondrial aggregation was unremarkable and autophagosomes could be observed (Fig. 3A).

Hippocampal microtubule–associated protein 1 light chain 3 (LC3) and sequestosome 1 (P62) mRNA showed an increasing trend in model mice, and the trend was reversed in mice treated with high-dose PNS. The ratio of LC3-II/I protein in the hippocampi of model mice was significantly low (*P* < 0.01), and treatment with high- and low-dose PNS considerably increased the LC3-II/I ratio (*P* < 0.01, compared with the model group; Fig. 3B-E).

### PNS activates the PINKI/Parkin pathway in the hippocampi of SAMP8 mice

**Fig. 4:**
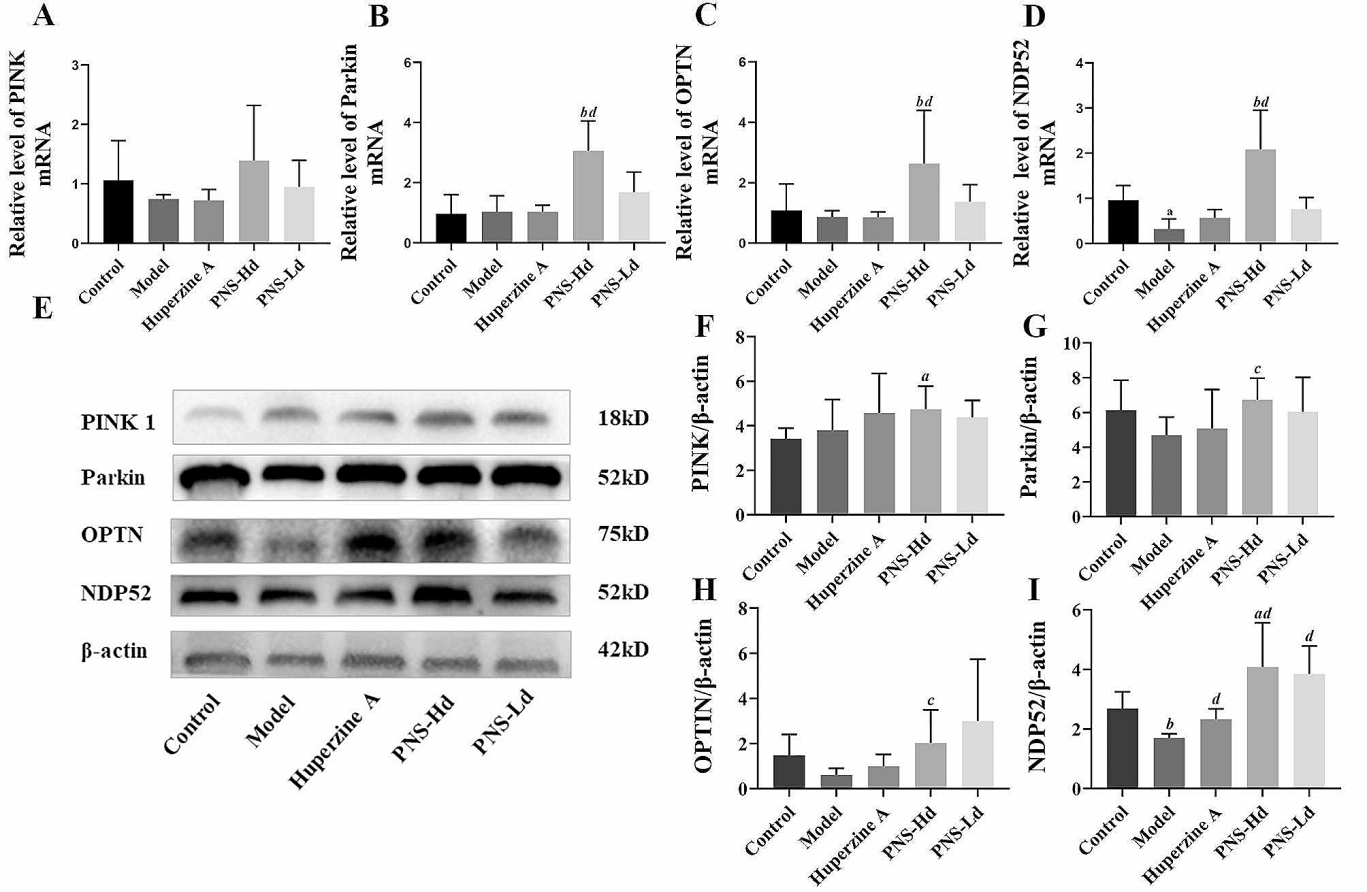
*Panax notoginseng* saponins activate the PINK1/Parkin mitochondrial autophagic pathway in hippocampal tissue of SAMP8 demented mice. **A-D** shows the effects of PNS on the expression levels of PINK1, Parkin, OPTN and NDP52 mRNAs, *n* = 9. **E** Western blot band plots of PINK1, Parkin, OPTN, and NDP52 proteins. **F-I** quantitative analysis of PINK1, Parkin, OPTN, and NDP52 protein expression, *n* = 5. ^*a*^*P* < 0.05, ^*b*^*P* < 0.01 compared with the control group, ^*c*^*P* < 0.05, ^*d*^*P* < 0.01 compared with the model group

The levels of PINK1, nuclear dot protein 52 (NDP52), and optineurin (OPTN) mRNA in the hippocampi of model mice were lower compared with those of mice in the control group. However, the levels of OPTN, NDP52, and Parkin mRNA in the hippocampi of mice treated with high -dose PNS were significantly higher (*P* < 0.01) than those in model mice (Fig. 4A-D). Though there is no significant difference between PINK1 mRNA levels in the model group and the control group, a tendency to increase PINK1 mRNA is also observed in high dose PNS-treated mice. The abundance of PINK1 protein in the hippocampi of model mice showed no significant differences from that in the controls (*P* > 0.05) but increased in the PNS-Hd and PNS-Ld groups. Parkin protein levels decreased in model mice compared with those in the controls; it was noteworthy that treatment with high-dose PNS significantly increased Parkin levels (*P* < 0.05). The abundance of NDP52 protein decreased significantly in model mice compared with that in the controls (*P* < 0.01), and the level of OPTN protein exhibited a decreasing trend (*P* > 0.05); however, treatment with high- and low-dose PNS induced a significant elevation in the levels of NDP52 and OPTN proteins (*P* < 0.05) (Fig. 4E-I). These results indicated that the PINKI/Parkin pathway, which regulates mitophagy, was activated in PNS-treated mice.

## Discussion

PNS are the main active ingredients of *P. notoginseng*, which is responsible for preventing cell death caused by neurotoxins such as Aβ. It alleviates neuronal dysfunction and has prophylactic and therapeutic effects in neurodegenerative diseases [42]. In this study, PNS was found to reduce oxidative stress in the brain of rapid aging SAMP8 mice, more critically, PNS increased Parkin and enhanced its recruitment to the mitophagic receptors, and further increased mitophagy in the hippocampi. These findings contribute novel evidence to enhance our comprehension of the mechanism of action of PNS in the treatment of AD, suggesting that modulating the mitophagy in the centurial nerves system may serve as a key mechanism underlying the therapeutic efficacy of PNS. The study also provides a theoretical basis for the clinical application of PNS formulations in age-related neurodegenerative diseases, such as AD.

Impaired mitochondrial function and insufficient cellular energy supply in patients with AD promote ROS formation, which initiates the peroxidation of membrane lipids and results in excessive fragmentation of mitochondria [43]. These ROS not only inflict damage upon proteins, membrane lipids, and nucleic acids, but also leads to disrupt cell signaling and prompt abnormal activation of transcription factors, consequently leads to the formation of Aβ plaques and neurofibrillary tangles in the brains of patients with AD [14]. Therefore, therapeutic interventions aimed at enhancing mitochondrial function and reducing oxidative stress can benefit AD patients. PNS enhances resistance to oxidative stress and thus alleviates Aβ-mediated neurotoxicity in *Caenorhabditis elegans* [27]. It also upregulates the expression of uncoupling proteins to protect neurons in the brains of AD mice to enhance mitochondrial defense against oxidative stress [33]. In the present study, our results suggest that PNS administration leads to an elevation in antioxidant enzyme activity a reduction in peroxide production, which suggesting a mitigating effect of PNS on oxidative stress in mouse brain. These findings were consistent with previous studies. Consequently, it can be posited that the amelioration of oxidative damage in brain tissue constitutes a significant mechanism through which PNS improves cognitive performance in SAMP8 mice.

Autophagy is a biological process by which cells clear damaged organelles and is regulated by a series of autophagy-related genes (Atg) [44]. When it occurs, Atg4 protease shears at the carboxyl terminus of newly synthesized LC3 to generate LC3-I, which subsequently binds to phosphatidylethanolamine through a ubiquitin-like reaction involving Atg3 and Atg7 to form lipidated LC3-II and attaches to the autophagosomal membrane surface [45]. Hence, LC3II is an early marker of intracellular autophagic activity [46], and the LC3-II/I ratio reflects the occurrence of cellular autophagy. In the present study, both LC3 mRNA levels and LC3-II/I ratio increased in PNS-treated mice, suggesting that PNS promoted cellular autophagy. Based on the nutritional status, autophagy was subdivided into nonselective autophagy under starvation conditions and selective autophagy under nutrient-rich conditions, such as mitophagy [47]. Mitophagy is essential for mitochondrial renewal and maintenance of function under physiological conditions. This process is highly activated in response to oxidative stress, mitochondrial transmembrane potential collapse, hypoxia, and iron starvation [48]. In cellular micromorphology, mitophagy is characterized by the presence of a double layer of membrane-like structures surrounding the mitochondria, or the tendency of the mitochondria to be wrapped. This wrapping process relies on the interaction of LC3 with the mitochondrial receptor proteins to promote bilayer formation, followed by autophagosomes wrapping around the damaged mitochondria [49, 50]. The hippocampal cells of mice in the model SAMP8 group showed severe mitochondrial damage with no autophagosomes. However, PNS treatment led to the autophagic vesicles to wrap around the mitochondria in hippocampus. Combined with elevated LC3 mRNA levels and increased LC3-II/I ratio, these findings demonstrate that treatment with PNS promotes the occurrence of mitophagy in the hippocampus of SAMP8 mice with dementia.

The PINK1/Parkin pathway is an important pathway that mediates mitophagy [51]. PINK1 is a protein kinase that monitors mitochondria, whereas Parkin acts as a ubiquitin-protein ligase that attaches ubiquitin chains to faulty mitochondria as a degradation signal [52]. PINK1 and Parkin synergistically promote the autophagic clearance of damaged mitochondria. PINK1 is rapidly degraded in healthy mitochondria after entering the inner mitochondrial membrane through the mitochondrial membrane complex. In damaged mitochondria, PINK1 is located in the outer mitochondrial membrane, where it recruits Parkin and promotes its phosphorylation. p-Parkin binds to the S65 site of the ubiquitin-like domain to ubiquitinate various membrane proteins on the mitochondrial surface, eventually promoting mitophagy [53]. Mitophagy dysfunction is a key mechanism in the cellular dysfunction and/or death in neurodegenerative diseases [54]. PINK1/Parkin regulates the initiation of mitophagy, which is crucial for mitochondrial quality control, potential interventions centered around their regulation of mitophagy are thought to offer new strategies for the treatment of neurodegenerative diseases [55]. PINK1 and Parkin mRNA levels were downregulated in the hippocampi of SAMP8 mice with dementia but were highly upregulated in PNS-treated mice. Additionally, the abundance of Parkin protein increased significantly, suggesting that PNS may activate the PINK1/Parkin pathway that regulates mitophagy. Autophagy receptor proteins such as NDP52 and OPTN are located downstream of the PINK1/Parkin and are recruited to the mitochondrial surface by ubiquitinated membrane protein ubiquitin chains that bind directly to LC3 through their LC3-interaction region. The binding facilitates the recruitment of autophagosomes to the mitochondria to mediate the autophagic clearance of damaged mitochondria [56, 57]. The response to Parkin recruitment of NDP52 and OPTN further amplifies the PINK1-triggered mitochondrial autophagic signal [58, 59]. Our study demonstrated the upregulation of NDP52 and OPTN mRNA in PNS-treated mice and a concomitant increase in their protein levels, suggesting that the significant increase in Parkin levels leads to substantial recruitment of the autophagic receptors NDP52 and OPTN, which is a sign of enhanced mitophagy.

Overall, PNS enhanced hippocampal mitophagy and attenuated oxidative stress in the brains of SAMP8 mice with dementia, These results were consistent with our previous findings of PNS enhancing mitophagy against Aβ-induced cellular damage in PC12 cells [39]. However, this study is subject to several imitations. First, in order to enhance the validity of our findings, it would be beneficial to assess mitochondrial membrane potential, oxygen consumption rate, oxidative phosphorylation, and ATP generation, as these factors are indicative of improved mitochondrial function resulting from enhanced mitophagy. Second, while the measurement of P62 at the gene level was conducted in this study, it is crucial to also determine its protein level in order to obtain a comprehensive understanding as an autophagy marker. Third, our animal experiments lacked further blocking experiments to fully validate the mitophagy promoting mechanism of PNS, and it is essential to carry out follow-up studies to explore the above issues in vivo. Furthermore, considering that the onset of senile dementia in SAMP8 mice is not identical to the pathological mechanism of AD caused by Aβ protein, it was speculated that the neuroprotective mechanism of PNS may not depend on the type of neurotoxin; it may, however, protect neuronal structure and function through autophagy-related mechanisms in neurodegenerative diseases. It is necessary to validate the effects of PNS in other neurodegenerative disease models with different pathologies to better elucidate the anti-dementia mechanism of PNS.

## Conclusions

PNS improved the learning and memory capacities of SAMP8 mice with dementia, cleared damaged mitochondria in the hippocampal, and attenuated oxidative stress in the brain. These effects may be closely related to enhancing mitophagy associated with the PINK1/parkin pathway and promoting the recruitment of Parkin to amplify mitophagic signaling. This could be one of its pathways for regulating mitochondrial homeostasis to improve cognitive impairment in neurodegenerative diseases.

### Electronic supplementary material

Below is the link to the electronic supplementary material.

**Supplementary Material 1:** Supplement figures of western blotting

## Data Availability

The data used to support the findings of this study are available from the corresponding author upon request.

## Abbreviations

8-OHdG: 8-hydroxy-2′-deoxyguanine
Aβ: Amyloid-beta
AD: Alzheimer’s disease
ATG: Autophagy related gene
ECL: Enhanced chemiluminescence
FDA: Food and Drug Administration
GSH-Px: Glutathione peroxidase
GSH: Glutathione
LC3: Microtubule-associated protein 1 light chain 3
MDA: Malondialdehyde
MWM: Morris water maze
NDP52: Nuclear dot protein 52
OPTN: Optineurin
PBS: Phosphate-buffered saline
PCR: Polymerase chain reaction
PINK1: PTEN-induced putative kinase 1
PNS: *Panax notoginseng* saponins
ROS: Reactive oxygen species
RT-qPCR: Reverse transcription-polymerase chain reaction
SAM: Senescence-accelerated mouse
SAMP: Senescence-accelerated mouse prone
SAMR: Senescence-resistant
SD: Standard deviation
SOD: Superoxide dismutase
