# Supplementary material for: Panax notoginseng saponins prevent dementia and oxidative stress in brains of SAMP8 mice by enhancing mitophagy
Source: BMC Complement Med Ther. 2024 Apr 4;24:144. doi: 10.1186/s12906-024-04403-7 (PMC10993618; doi:10.1186/s12906-024-04403-7)
Supplement: Supplementary file 1 — Supplementary Material 1: Supplement figures of western blotting [file 12906_2024_4403_MOESM1_ESM.pdf]

LC3

Raw Picture in Fig. 3E, the blue and red box position indicates the cutting position and both blots were running on the same gel.

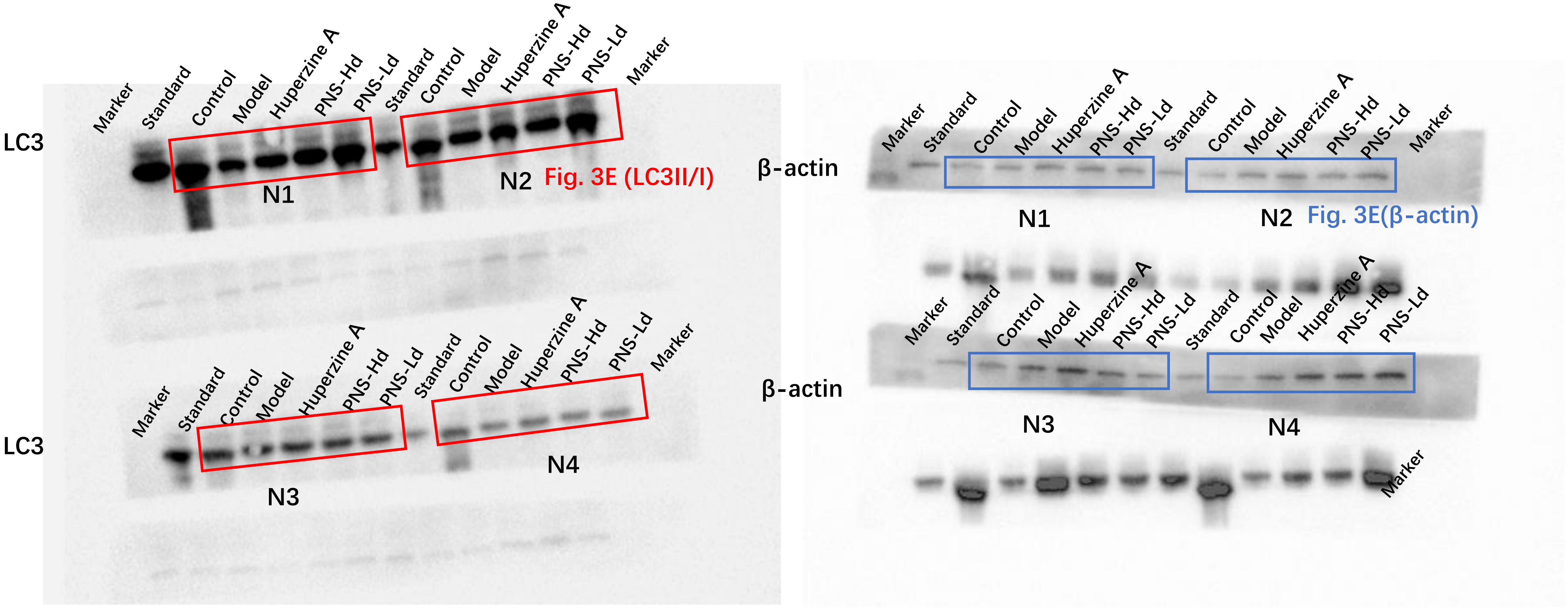

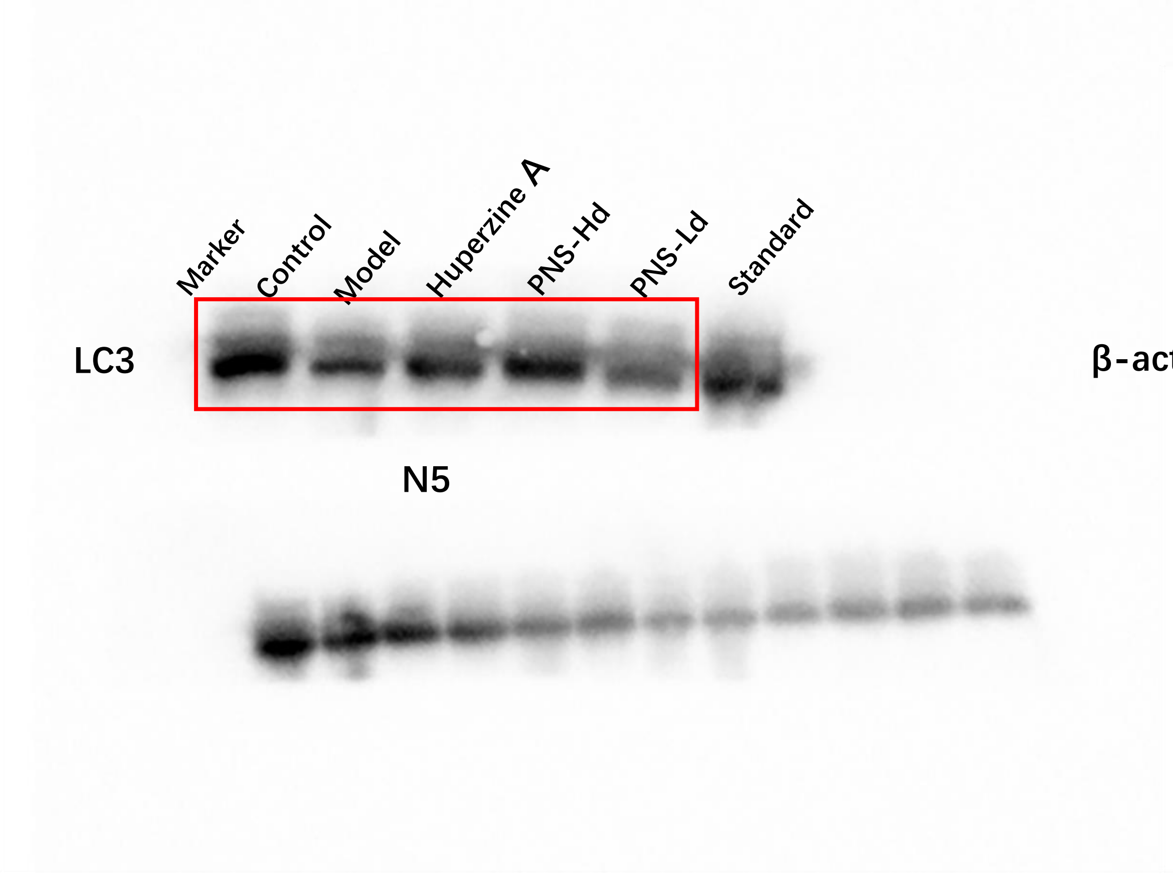
Marker
Control
Model
Huperzine A
PNS-Hd
PNS-Ld
Standard
LC3
N5

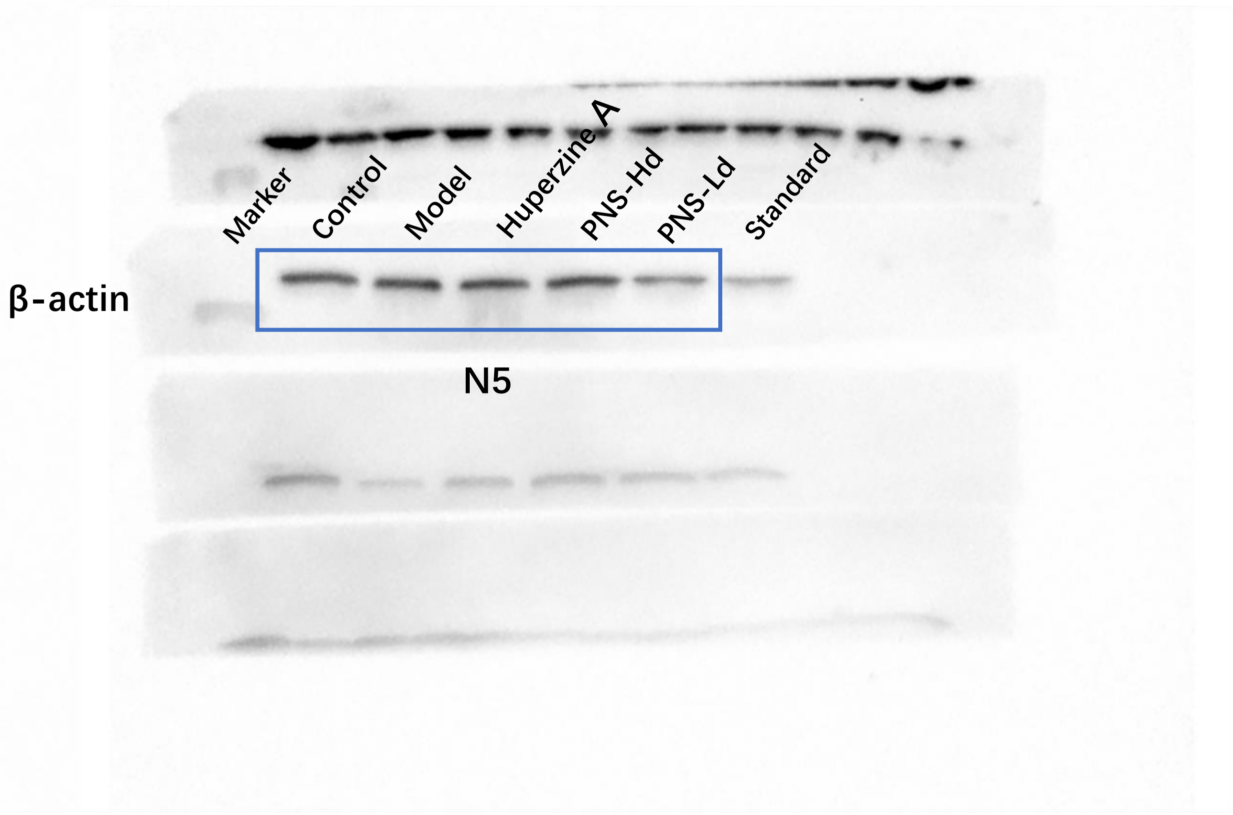
Marker
Control
Model
Huperzine A
PNS-Hd
PNS-Ld
Standard
β-actin
N5

Parkin

Raw Picture in Fig. 4E, the blue and red box position indicates the cutting position and both blots were running on the same gel.

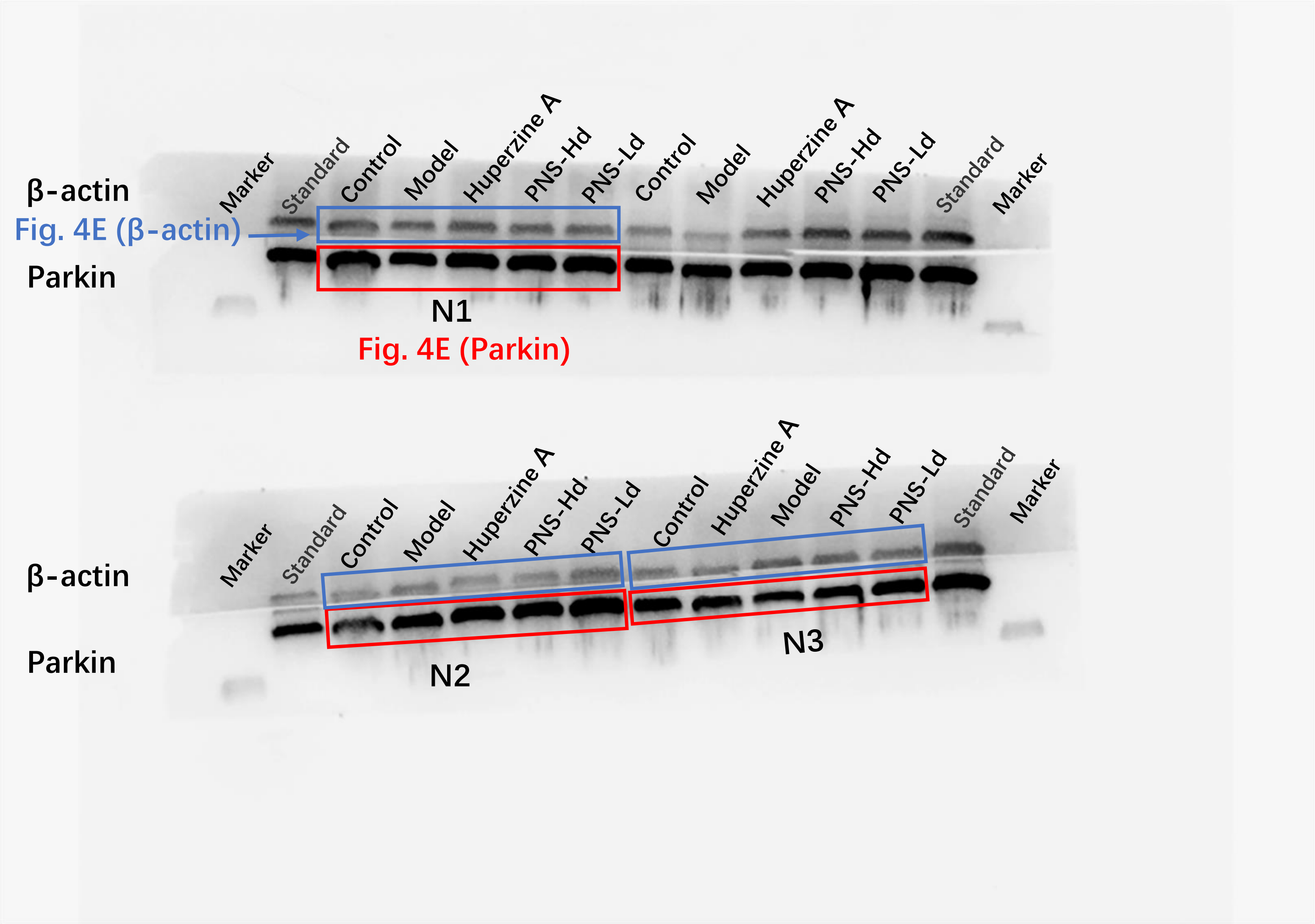

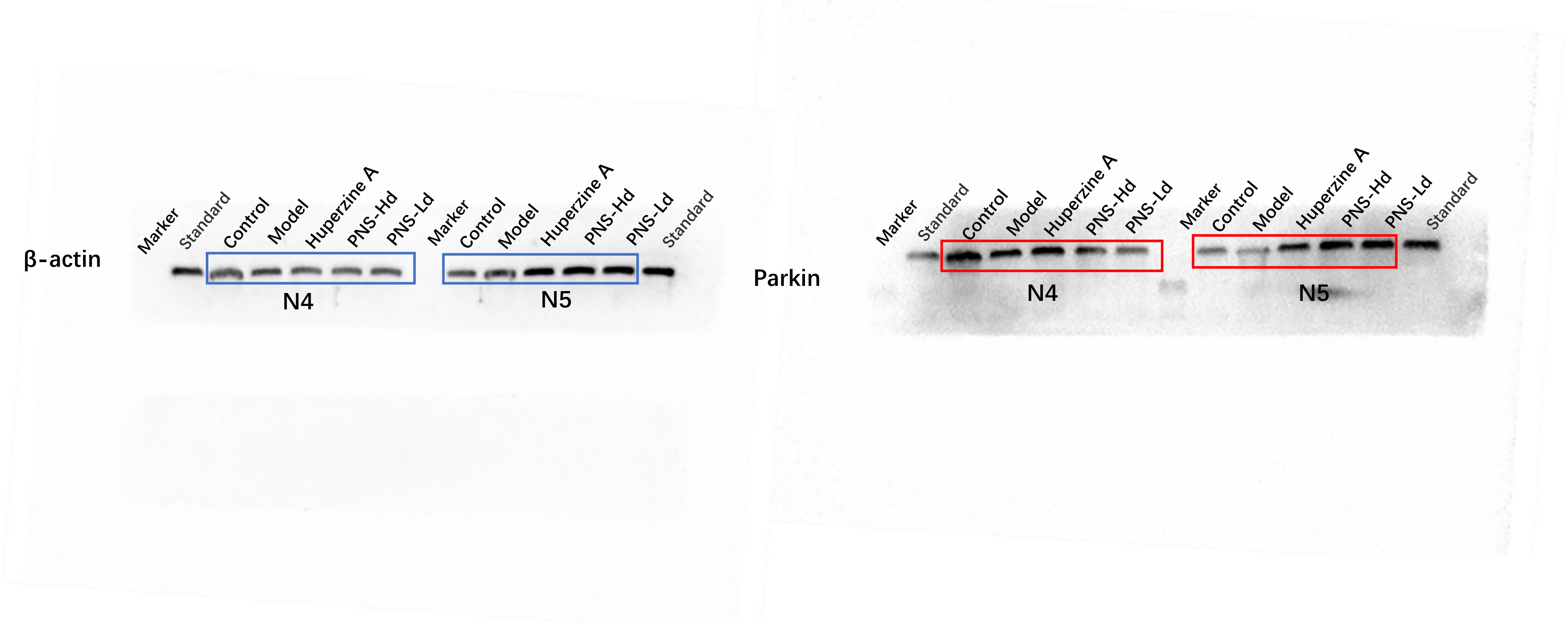

β-actin
Marker
Standard
Control
Model
Huperzine A
PNS-Hd
PNS-Ld
Marker
Control
Model
Huperzine A
PNS-Hd
PNS-Ld
Standard
N4
N5
Parkin
Marker
Standard
Control
Model
Huperzine A
PNS-Hd
PNS-Ld
Marker
Control
Model
Huperzine A
PNS-Hd
PNS-Ld
Standard
N4
N5

PINK 1

Raw Picture in Fig. 4E, the blue and red box position indicates the cutting position and both blots were running on the same gel.

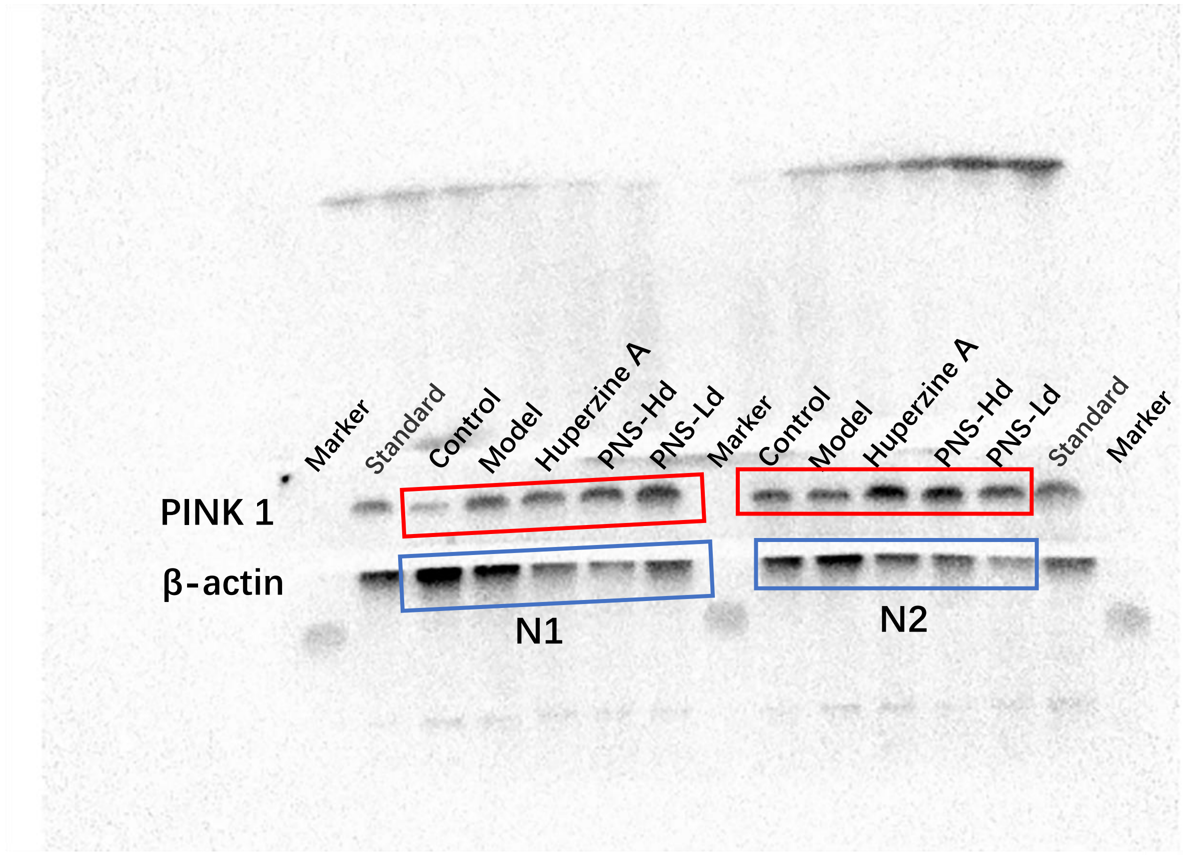

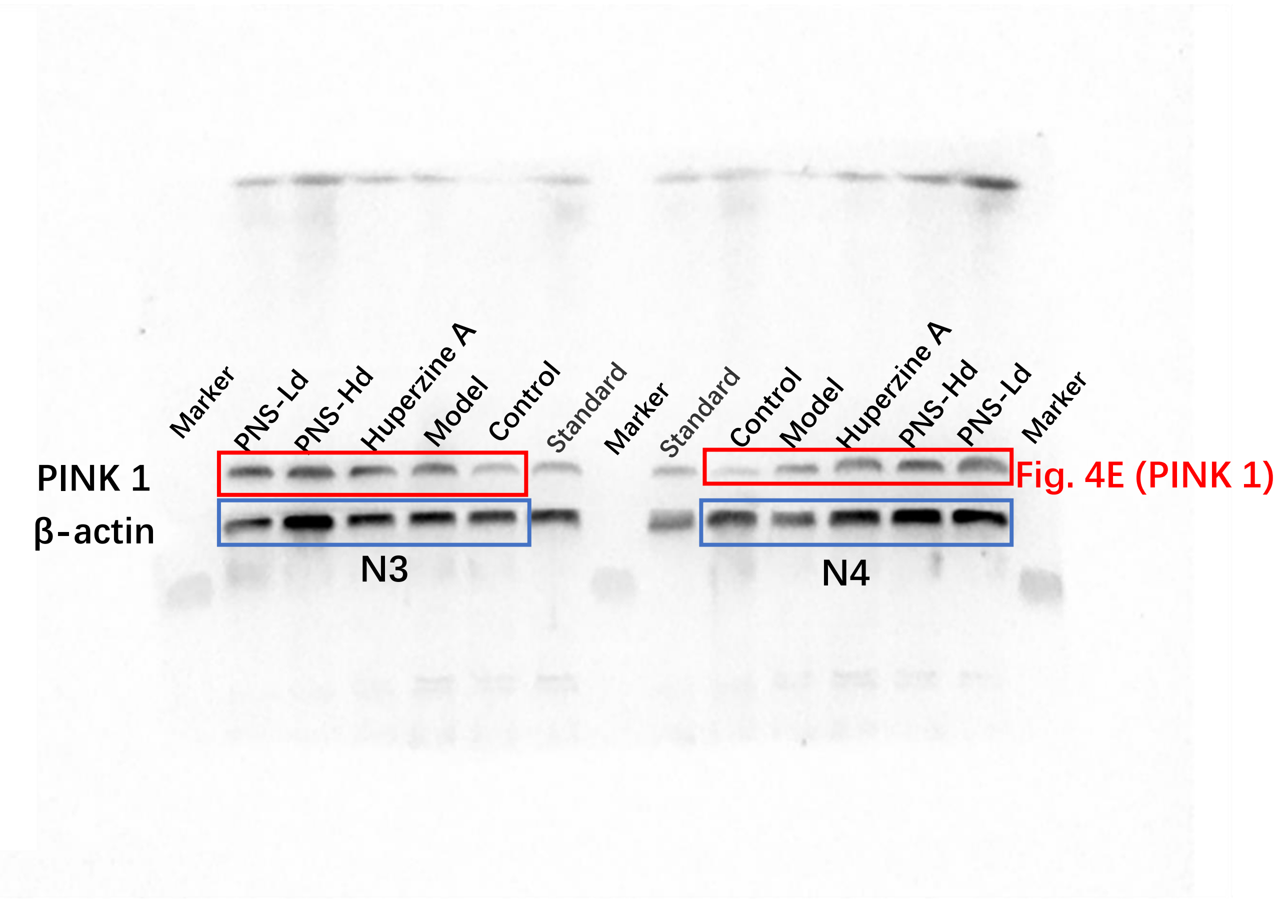

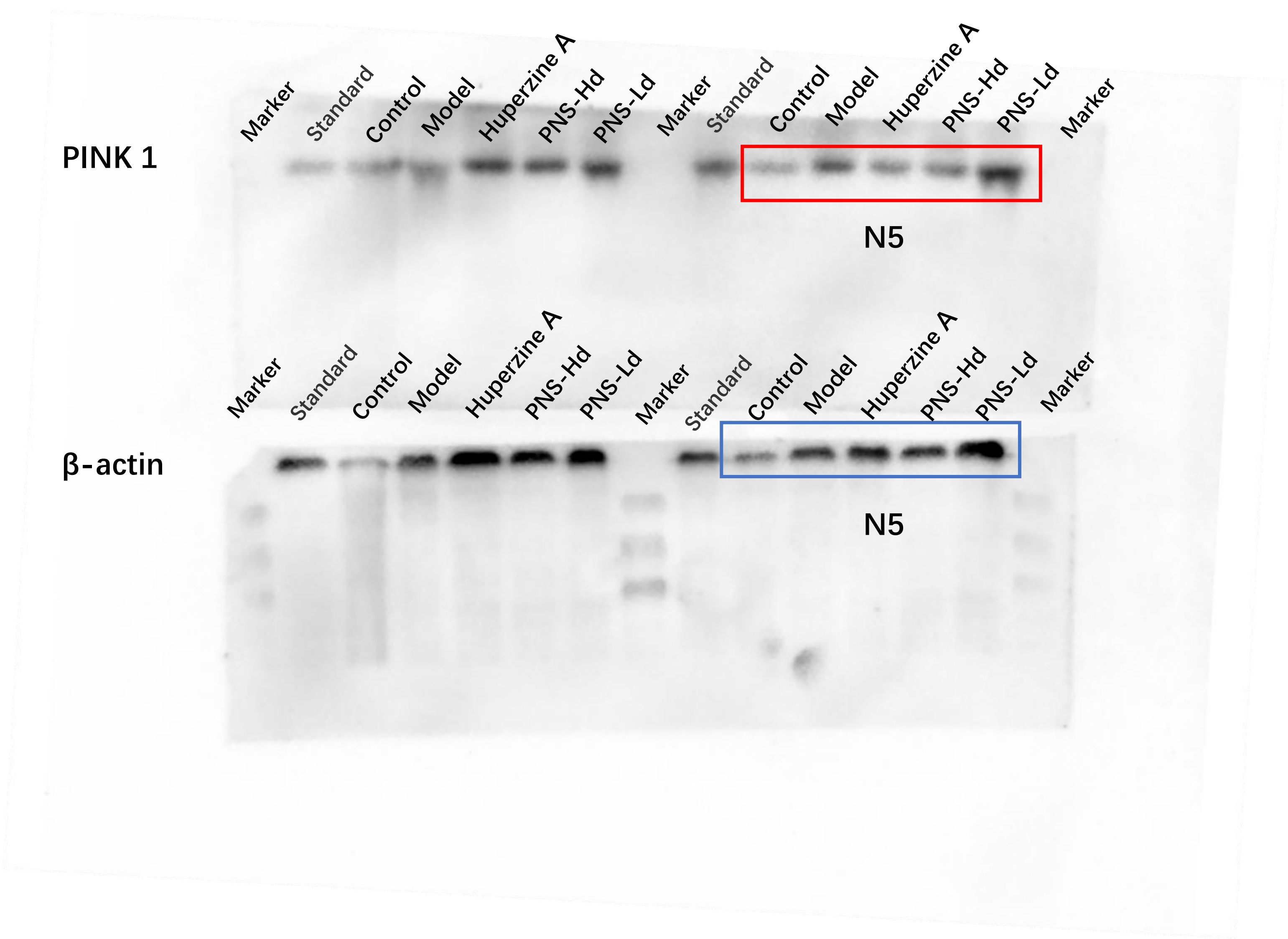
PINK 1
Marker
Standard
Control
Model
Huperzine A
PNS-Hd
PNS-Ld
Marker
Standard
Control
Model
Huperzine A
PNS-Hd
PNS-Ld
Marker
N5
β-actin
Marker
Standard
Control
Model
Huperzine A
PNS-Hd
PNS-Ld
Marker
Standard
Control
Model
Huperzine A
PNS-Hd
PNS-Ld
Marker
N5

OPTN

Raw Picture in Fig. 4E, the blue and red box position indicates the cutting position and both blots were running on the same gel.

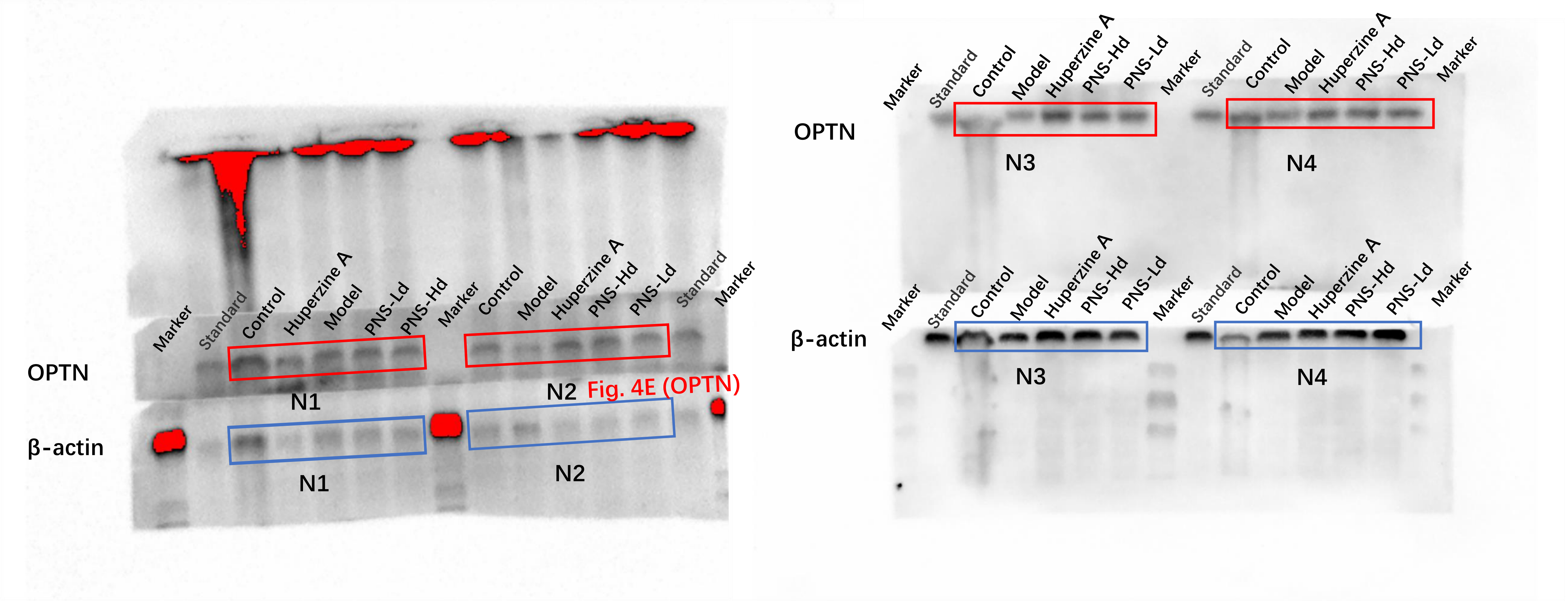

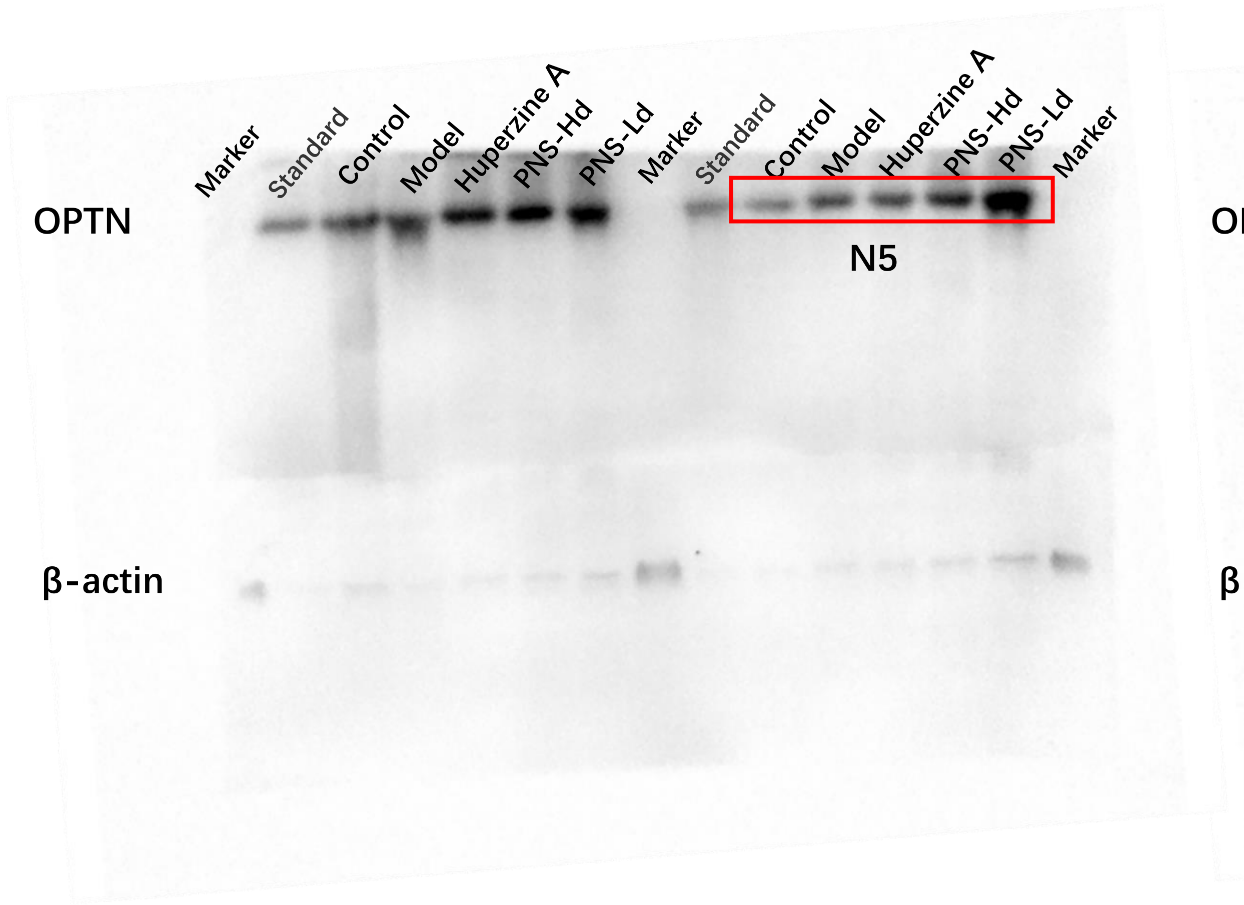
Marker
Standard
Control
Model
Huperzine A
PNS-Hd
PNS-Ld
Marker
Standard
Control
Model
Huperzine A
PNS-Hd
PNS-Ld
Marker
OPTN
N5
β-actin

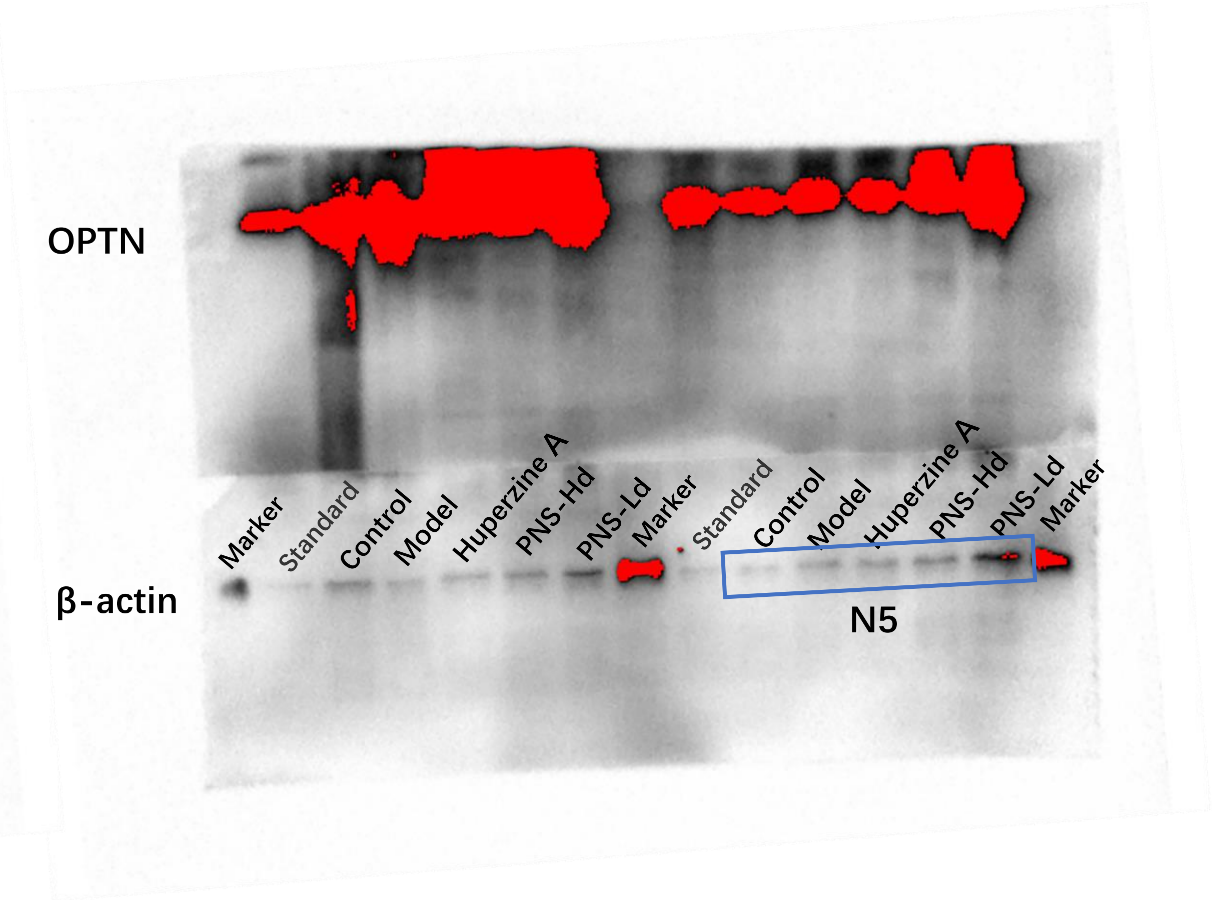
OPTN
Marker
Standard
Control
Model
Huperzine A
PNS-Hd
PNS-Ld
Marker
Standard
Control
Model
Huperzine A
PNS-Hd
PNS-Ld
Marker
N5
β-actin

NDP52

Raw Picture in Fig. 4E, the blue and red box position indicates the cutting position and both blots were running on the same gel.

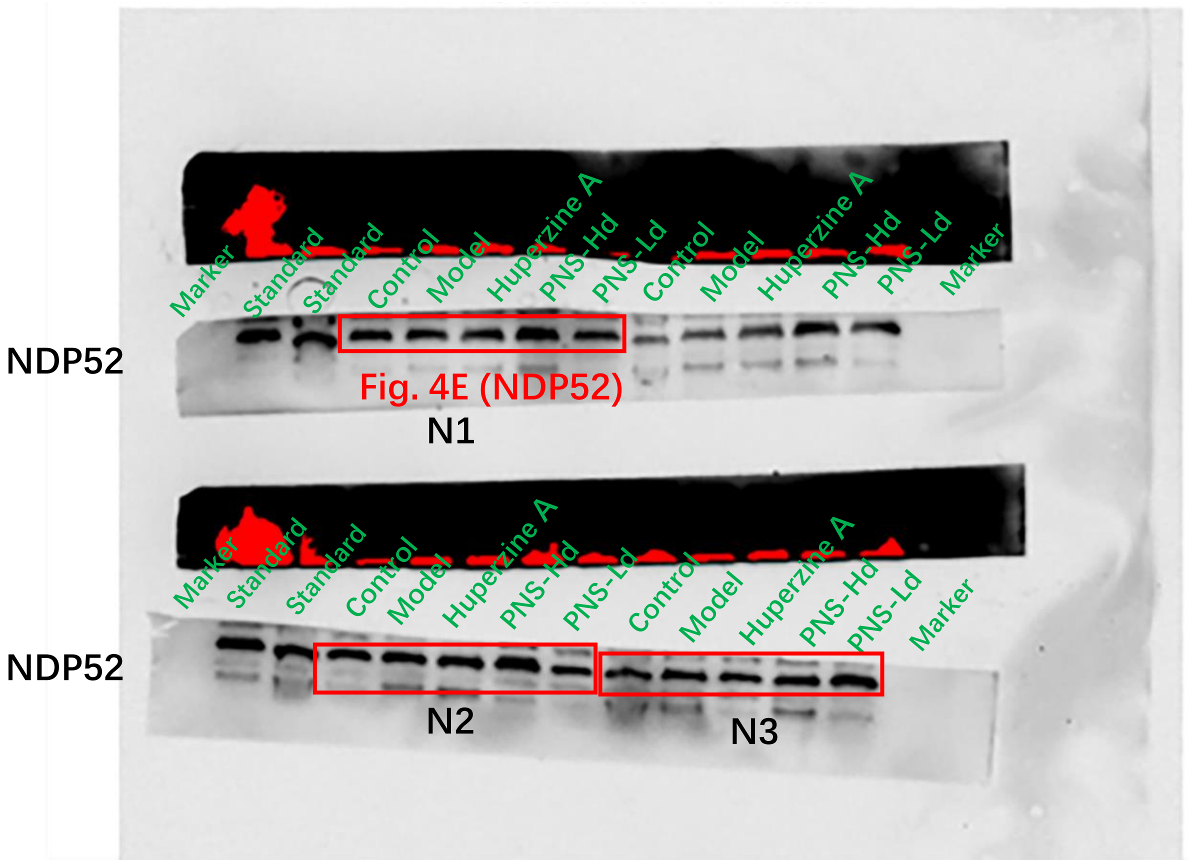

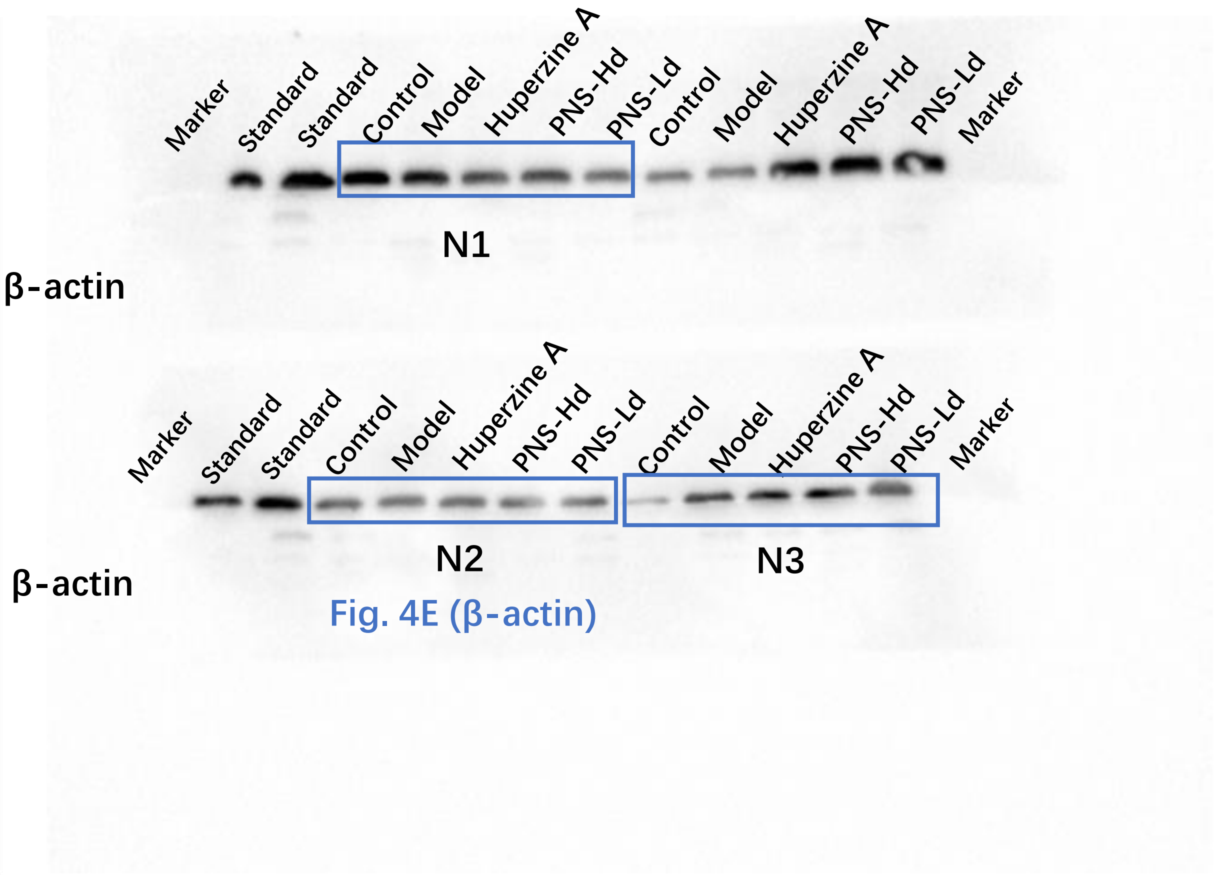

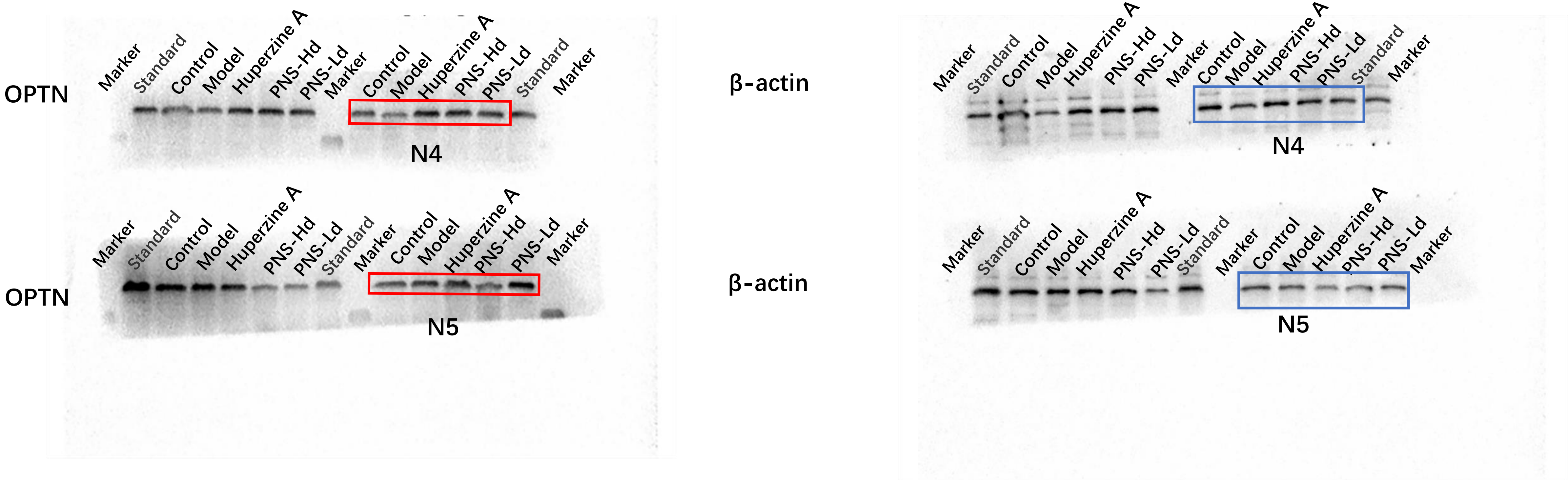
OPTN
Marker Standard Control Model Huperzine A PNS-Hd PNS-Ld Marker Control Model Huperzine A PNS-Hd PNS-Ld Standard Marker
N4
OPTN
Marker Standard Control Model Huperzine A PNS-Hd PNS-Ld Standard Marker Control Model Huperzine A PNS-Hd PNS-Ld Marker
N5
β-actin
Marker Standard Control Model Huperzine A PNS-Hd PNS-Ld Marker Control Model Huperzine A PNS-Hd PNS-Ld Standard Marker
N4
β-actin
Marker Standard Control Model Huperzine A PNS-Hd PNS-Ld Standard Marker Control Model Huperzine A PNS-Hd PNS-Ld Marker
N5
